# Transcatheter aortic valve implantation against conventional aortic valve replacement surgery in high-risk patients with aortic stenosis; a cost-effectiveness analysis

**DOI:** 10.1186/s13561-022-00411-w

**Published:** 2023-01-03

**Authors:** Hesam Ghiasvand, Shiva Khaleghparast, Naser Kachoueian, Kourosh Tirgarfakheri, Meysam Mortazian, Yaser Toloueitabar, Farhad Gorjipour, Seyran Naghdi

**Affiliations:** 1 Division of Health Sciences, Warwick Medical School, University of Warwick, Coventry, UK; 2 Cardiovascular Nursing Research Center, Rajaie Cardiovascular Medical and Research Center, Iran University of Medical Sciences, Tehran, Iran; 3 Department of Cardiac Surgery, Imam Hossein Educational Hospital, Shahid Beheshti University of Medical Sciences, Tehran, Iran; 4 Rajaie Cardiovascular Medical and Research Center, Iran University of Medical Sciences, Tehran, Iran; 5 AJA University of Medical Sciences Tehran Iran AJA University of Medical Sciences, Tehran, Iran; 6 Iranian Scientific Society of Extracorporeal Technology, Rajaie Cardiovascular Medical and Research Center, Iran University of Medical Sciences, Tehran, Iran; 7 National Center for Health Insurance Research, Tehran, Iran

**Keywords:** Aortic stenosis, Transcatheter Aortic Valve Implantation (TAVI), Surgical Aortic Valve Replacement, Cost-effectiveness analysis, Economic evaluation

## Abstract

**Background:**

Aortic stenosis is a prevalent heart valvular disorder in Iran. Transcatheter Aortic Valve Implantation (TAVI) and Surgical Aortic Valve Replacement (SAVR) are two common procedures for treating the disease in the current clinical pathway. However, TAVI is an expensive procedure, and for Iran with severe limitations in financial resources, it is crucial to investigate the cost-effectiveness of the technology against other competing alternatives with the same purpose. This study aims to analyse the cost-effectiveness of TAVI vs SAVR in elderly patients who are at a higher risk of surgery.

**Methods:**

This study is a decision economic evaluation modeling, with a lifetime horizon and a healthcare payer (health insurer) perspective. The utility values are from a previous study, transitional probabilities come from an established clinical trial called PARTNER-1, and the unit costs are from Iran’s national fee schedule for medical services. The probabilistic and one-way sensitivity analyses have been performed to mitigate the uncertainty.

**Results:**

The incremental cost, effectiveness, and cost-effectiveness ratio for the base case were: 368,180,101 Iranian Rial, (US$ 1,473), 0.37 QALY-per-patient, and, 995,081,354 Iranian Rial (US$ 3,980), respectively. The probabilistic sensitivity analysis yielded 981,765,302 I.R.I Rials (US$ 3,927) per patient for the ICER. The probability of being cost-effective at one and three times the country’s Gross Domestic Production (GDP) is 0.31 and 0.83, respectively.

**Conclusions:**

TAVI does not seem a cost-effective procedure in comparison with SAVR at the current willingness to pay thresholds of the country. However, by increasing the WTP threshold to 3 times the GDP per capita the probability of being cost-effective will raise to 83%.

## Introduction

Cardiovascular diseases (CVDs) are among the top mortality and morbidity causes, globally. During recent decades, the number of lost years by either disability or deaths attributed to CVDs has increased [1]. Heart valvular disorders (HVDs) including mitral and aortic valves have a direct association with the growing aged population around the world [2]. Aortic Stenosis (AS) is a prevalent heart valvular problem. The progression of the disease is greater among the aged population [3].

The disease can increase the risk of death between 25 to 50% at the severe and symptomatic phases. The risk of death can also potentially increase to 90% in five years among those groups of patients who haven’t received appropriate therapeutic actions. It also harms the patients' quality of life (QoL) [4]. The findings of the quality of life at the symptomatic phase of AS showed that patients are faced with a significant decrease in their physical and social functions. Among elder AS patients (above 65 years) emotional functions, vitality, and mental health have been also degenerated significantly in comparison with the general population [5].

AS is among diseases with notable direct and indirect costs impact on health systems. The results of a study in the United States showed that symptomatic AS can cause an incremental effect of $12,789 on the annual per-patient healthcare expenditure. At the asymptomatic phase, this incremental effect is $10,816. The annual total attributed costs to symptomatic and asymptomatic AS have been $ 5.6, and $4.6 billion, respectively [6].

Surgical Aortic Valve Replacement (SAVR) and Transcatheter Aortic Implantation (TAVI) are two commonly recommended interventions for treating AS. SAVR is an invasive open-heart surgery for replacing mechanical or biological prostheses with regurgitating aortic valves. TAVI is known as a minimally invasive procedure (MIP) that takes benefits from technological advancement to replace the broken aortic valve [7, 8]. The evidence supports the positive impact of Aortic Valve Replacement (AVR) on the improvement of QoL, and cardiac symptoms among elderly groups [9].

The economic evaluation evidence supports the cost-effectiveness of TAVI as an alternative to SAVR among aged patients who might be at a higher risk for conventional surgery. This evidence is mainly from developed countries including the USA, United Kingdom, Japan, France, Belgium, and Canada. For these high-income countries with a considerable proportion of the senior population, TAVI seems to be a promising procedure that could be cost-effective [9–11]. This evidence has used the results of a large Randomized Controlled Trial which is called PARTNER-1 which enrolled the patients at higher risk of operative procedure [12]. The cost-effectiveness of TAVI in recent years has been also investigated for the population at the intermediate and low-risk of surgery due to expanding PARTNER-1 and targeting the intermediate and low-risk patients, as well [13–15].

In Iran, SAVR is the more common procedure in managing AS, and it is benefited from the country’s basic health insurance obligations. Cardiovascular disease is the leading cause of death and disability-adjusted life-years in the country. It is also assumed that the prevalence of AS will be growing as the country’s population is aging [16]. TAVI has been introduced to clinical practice in both public and private healthcare providers in the country. However, as a lower-middle-income country according to the World Bank classification in 2020, the policymakers must know if a technology such as TAVI is cost-effective to be considered in the national health benefit package. Therefore, the purpose of this study is to compare the cost-effectiveness of TAVI with SAVR in managing high-risk elder patients.

## Methods

The study follows the Consolidated Health Economic Evaluation Reporting Standards 2022 (CHEERS 2022) statement format [17].

### The setting, and population

The study was conducted for Iran as a lower-income country according to the World Bank income classification system in 2020 [18]. Currently, the main financial stream for the public/governmental health care provision is public mandatory health insurance which is restricted in terms of cost-sharing [19]. At present, there is not any public health insurance benefit for the TAVI procedure, and it is paid through the patients’ out-of-pocket payments or private insurance schemes. Patients who have aortic stenosis aged 75 years or older with a higher risk of sugary undergo either TAVI or SAVR as the intervention and comparator, respectively. We assumed the populations are similar in terms of their underlying health problems.

### Model structure

We developed a hybrid model including a decision tree for the first thirty days post-procedures and then a Markov model with a lifetime horizon. In the model structure patients who survived are categorised in one of the four (from I (mild illness) to IV (severe illness)) New York Heart Association (NYHA) classification bands and enter the longer-term monitoring and management pathway. In the longer-term model, patients possibly are experiencing different complications, but based on the clinical advice from the study’s clinical members we assumed the major stroke was the main and disabling complication alongside the NYHA bands. This advice is supported by other evidence as well [14, 20]. Therefore, for the Markov part of the model, we considered nine health states: four health states were indicated by NYHA-I to NYHA-IV; another four attributed the major stroke alongside the NYHA bands and ultimately the death state. The probabilities of transition between each health state to death are assumed identical.

**Fig. 1:**
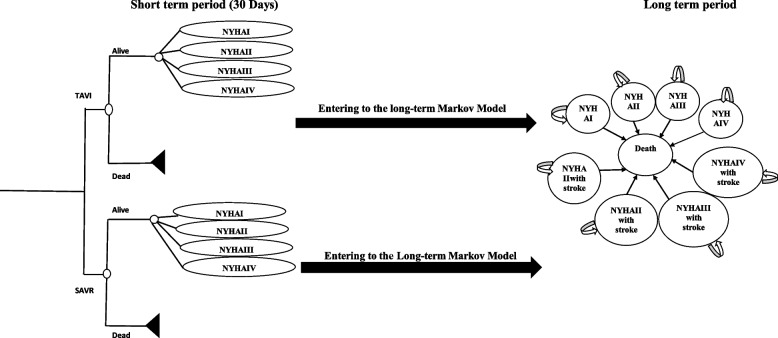
The decision-making model structure

Figure 1 presents the illustrative structure of the model. We conducted the analyses from the perspective of the third-part-payer (here health insurers). Both costs and effects were discounted by the same rate (6.5%) [21].

### Model inputs

#### Clinical effectiveness inputs

The data for rates of mortality, rehospitalization, major stroke, myocardial infarction, new atrial fibrillation, and new peacemaker implantation was extracted from a relevant randomised controlled trial (PARTNER-I) [14]. PARTNER-I is a large multicentre randomised controlled trial in three countries: the USA (23 centers), Germany (1 center), and Canada (2 centers). The trial compares the clinical outcomes of TAVI vs SAVR for managing AS among higher-risk patients. The trial enrolled 699 patients 348 assigned to TAVI, and 351 assigned to SAVR). The mean (SD) age of the TAVI arm was 83.6(6.8), and 84.5(6,4) for the SAVR arm. Most participants were male (57.8% TAVI, and 56.7% SAVR). All patients had severe symptomatic native trileaflet severe degenerative AS with a mean echocardiographic gradient ≥ 40 mm Hg or jet velocity > 4.0 m/s and an aortic valve area ≤ 0.8 cm2 [14, 20]. The researchers tailored the study for five years to capture the differences between TAVI and SAVR in terms of clinical outcomes including all causes mortality rate, cardiovascular causes mortality rate, and hospital re-admission rate [20]. We found a systematic review aimed to compare the effectiveness of TAVI against the SAVR for patients with severe AS. The study has used the quality appraisal based on the Cochrane risk of bias tool for RCTs. According to this study’s results, PARTNER-I has a high level of risk of bias in four domains including participants, personnel blinding, selective reporting, and other biases. The trial was assessed as “unclear” in terms of allocation concealment and blinding of assessors. However, the risk of bias is categorised as low for randomisation and incomplete outcome data. PARTNER-I is categorised at a high-level risk of bias in the mentioned review [22].

The utility weights by the NYHA classification system were adopted from Holland R., et al. (2010) [23]. These utility weights measure the quality of life for each NYHA class by EQ-5D. As the major stroke is a prevalent serious event, we assumed a disutility weight equates to 0.39 for the major stroke. This disutility was applied to the model after the first cycle of the longer-term model (Markov part) as we considered the contribution of the rehabilitation services to the patient's quality of life and its benefits for up to one year postprocedure [24].

#### Resources utilisation and costing sources

The costs include all relevant direct costs that are incurred by the health care payers (health insurers). It includes an inventory of attributed costs to delivering the procedures, and other related costs to the common complications (bleeding, stroke, myocardial infarction, and kidney failure are known as the most prevalent post procedures complications). The costs were adopted from the Iranian national fee schedules for the medical procedures that are available based on the Relative Value Units (RVU) schedule in the country. All cost is based on the Iranian Rial for 2020, we also converted total cost, cost-per-patient, and cost-per-QALYs to the US$ for the same year (1 US$ = 250,000 IR Rials on average in 2020).

### Characterizing uncertainty

We performed a probabilistic sensitivity analysis through the Monte Carlo method. In this method, the quantities attributed to each parameter (costs, QALYs, transition probabilities between health states…) can change in defined cumulative distribution functions at the same time. Therefore, it gives a robust method to mitigate the uncertainty surrounding the parameters and then the cost-effectiveness ratio.

In addition, we conducted a one-way sensitivity analysis to capture the impacts of contextual differences in terms of health outcomes and adverse event rates between Iran (a lower-middle-income country) and the source of the data (multicentre RCT in three high-income countries).

In addition, we used a Cost-Effectiveness Acceptability Curve (CEAC) for showing the probability of being cost-effective of TAVI at a range of willingness to pay thresholds. The willingness to pay presents the maximum money that a healthcare payer (health insurer) would like to pay to gain healthier states for the covered population. As in Iran, there is no explicit willingness to pay, we used the World Health Organization (WHO) criteria:

An intervention/service is cost-effective if the cost per each unit of health outcome (here QALYs) lies within a range between less than Gross Domestic Production (GDP) Per capita, to three times GDP per capita [25]. For Iran, the threshold range was between 551,400,000 (US$ 2,205) to 1,654,200,000 (US$ 6,075) IR. Iran's Rials equate to one to three times the country's GDP per capita in 2020 [26]. All calculations and analyses have been done in MS Excel.

#### Model parameters

Tables 1, 2 and 3 present the parameters for the model. The data for quality of life by NYHA classes was obtained from Holland, R. et al. [23] Table 1 depicts the utility values and associated standard deviations per each NYHA class.

**Table 1:** Model parameters for the quality of life of patients

Utility item	Base case	Standard Deviation	Distribution of interest	Source
Quality of Life (NYHA I)	0.72	0.25	Beta	[23]
Quality of Life (NYHA II)	0.72	0.25	Beta	[23]
Quality of Life (NYHA III)	0.53	0.32	Beta	[23]
Quality of Life (NYHA IV)	0.47	0.35	Beta	[23]
Disutility due to Major Stroke	0.39	–	–-	[24]

**Table 2:** Model parameters for the rate of risks and adverse events attributed to strategies

Rate of adverse events and Probability of transition	Base Case	Source
Transient ischemic attack (TIA) TAVI	0.053	[20]
Transient ischemic attack (TIA) SAVR	0.043	[20]
Stroke (TAVI)	0.153	[20]
Stroke (SAVR)	0.125	[20]
Rehospitalization (TAVI)	0.333	[20]
Rehospitalization (SAVR)	0.252	[20]
Myocardial infarction (TAVI)	0.111	[20]
Myocardial infarction (SAVR)	0.082	[20]
New atrial fibrillation (TAVI)	0.158	[20]
New atrial fibrillation (SAVR)	0.304	[20]
New permanent pacemaker implantation (TAVI)	0.155	[20]
New permanent pacemaker implantation (SAVR)	0.13	[20]
Aortic-valve reintervention (TAVI)	0.032	[20]
Aortic-valve reintervention (SAVR)	0.008	[20]
Transient ischemic attack (TIA) TAVI	0.053	[20]
Transient ischemic attack (TIA) SAVR	0.043	[20]
Probability of being at NYHA I) TAVI (0–12 months)	0.36	[14]
Probability of being at NYHA II) TAVI (0–12 months)	0.28	[14]
Probability of being at NYHA III) TAVI (0–12 months)	0.10	[14]
Probability of being at NYHA IV) TAVI (0–12 months)	0.01	[14]
Probability of death for any causes TAVI (0–12 months)	0.25	[14]
Probability of being at NYHA I) SAVR (0–12 months)	0.34	[14]
Probability of being at NYHA II) SAVR (0–12 months)	0.30	[14]
Probability of being at NYHA III) SAVR (0–12 months)	0.07	[14]
Probability of being at NYHA IV) SAVR (0–12 months)	0.02	[14]
Probability of death for any causes SAVR (0–12 months)	0.27	[14]

**Table 3:** The unit cost associated with TAVI and SAVR in current Iran’s clinical pathway for AS treatment

cost Item^*^	Mean cost^*^	Standard Deviation	Distribution of Interest	Source
SAVR costs of the procedure	12,811,000 (51)	–-		[27]
TAVI costs of the procedure	20,052,000 (80)	–-		[27]
TAVI costs of hospitalization	43,200,000 (173)	4,032,654	Gamma	[28]
SAVR costs of hospitalization	75,750,000 (303)	4,326,731	Gamma	[28]
Stent costs TAVI	450,000,000 (1,800)	–-		[27]
Valve Costs	800,000,000 (3,200)	–-		[27]
Permanent Peace Maker Implantation Procedure or Implantable cardioverter defibrillators (ICDs) Cost	9,183,500 (37)	–-		[27]
Cost of Angioplasty	7,398,300 (29)	–-		[27]
Reoperation TAVI	10,026,000 (40)	1,430,401	Gamma	[27]
Reoperation SAVR	6,405,500 (26)	2,720,528	Gamma	[27]
CABG	22,280,000 (89)	–-		[27]

*All costs are Iranian Rial (US$ equivalent)

The second part of the model inputs is related to the transition probabilities between different health states, and the risk of adverse events related to interventions.

The healthcare resource utilization costs are the final part of the model inputs. Table 3 provides the related data for the resource utilization part of the model.

### Main results

The base case analysis yielded an incremental cost of 368,180,101 I.R.I Rials (US$ 1,473) and 0.37 as incremental QALYs. These differences make a 995,081,354 I.R.I Rials (US$ 3,980) cost per patient. Table 4 presents the results of the base case scenario.

**Table 4:** base case analysis of the cost-effectiveness of TAVI vs SAVR

	Costs (I.R.I Rials)	QALYs	Incremental Cost	Incremental QALYs	ICER
TAVI	720,257,876 (US$ 2,881)	1.27	368,180,101 (US$ 1,473)	0.37	995,081,354 (US$ 3,980)
SAVR	352,077,775 (US$ 1,408)	0.90	–-	–-	–-

### Effects of uncertainty

**Fig. 2:**
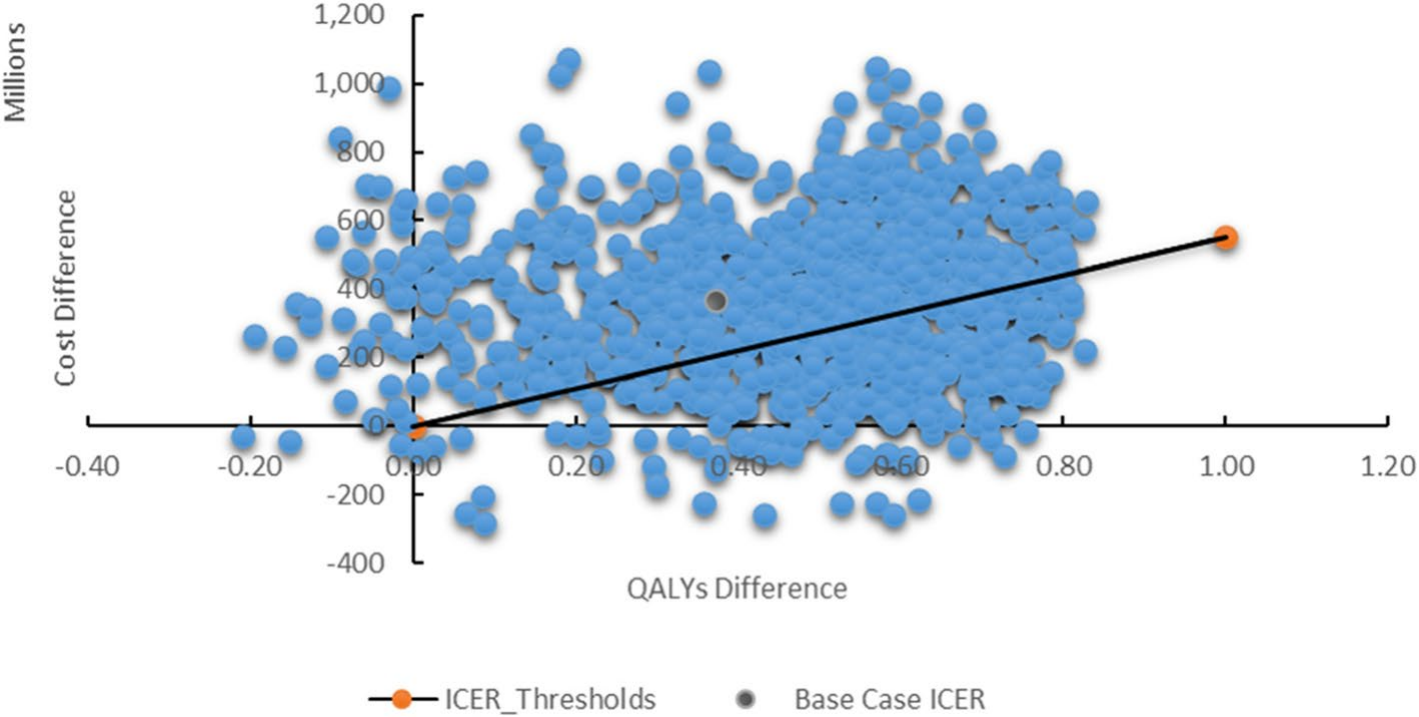
The cost-effectiveness scatter plane for TAVI vs SAVR

**Fig. 3:**
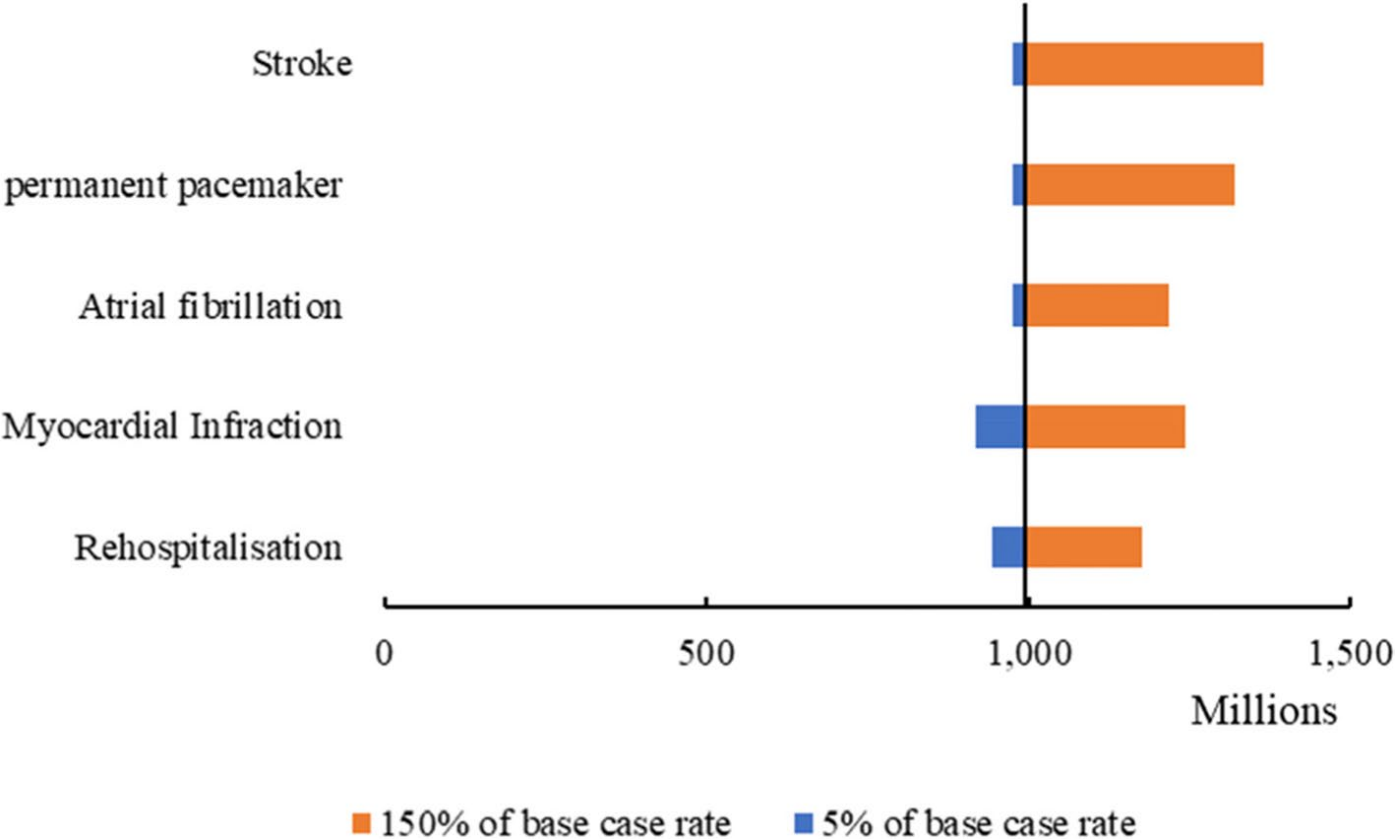
Tornado graph for one-way-sensitivity analysis

**Fig. 4:**
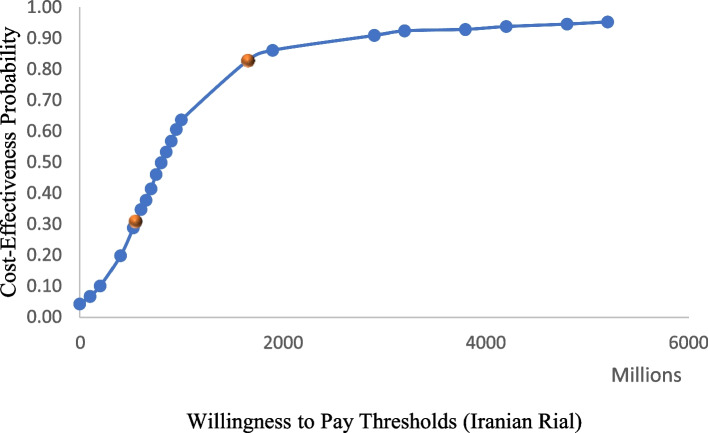
Cost-Effectiveness Acceptability Curve of TAVI vs SAVR

Figures 2, 3 and 4 show the results for mitigating the uncertainty around the model parameters. Figure 2 shows a scatter plot for the probabilistic sensitivity analysis result. The mean of ICER for the probabilistic sensitivity analysis is 981,765,302 I.R.I Rials (US$ 3,927) per patient.

#### One-way sensitivity analysis

Figure 3 shows the tornado plot for the initial model inputs that have had the most impact on the base case ICER in a range of 5 to 150% changes in their rates. Increasing the stroke rate has resulted in a 37% increase in the base case ICER which equates to 1,365,982,532 Iranian Rial (US$ 5,464). A 150% growth in the permanent pacemaker causes a 35% increase in base case ICER (1,320,762,751 Iranian Rial equates to US$ 5,238). If myocardial infarction grows up by 150%, the base case ICER will rise by 25% which means 1,243,862,843 Iranian Rial (US$ 4,975). Also, an 150% increase in Atrial Fibrillation can change the base case ICER by about 22% which will be 1,216,768,043 Iranian Rial (US$ 4,867). Finally, at a 150% change in the rehospitalization rate, the base case ICER will increase by about 18% and the ICER turns to 1,175,435,431 Iranian Rial (US$ 4,702).

The last piece of the study finding is showing the probability of cost-effectiveness of TAVI against SAVR at WTP thresholds. Figure 4 provides the graph of the cost-effectiveness analysis acceptability curve (CEAC) that presents the probability of cost-effectiveness for TAVI vs SAVR at different willingness to pay thresholds based on the country’s GDP per capita.

The CEAC shows that at the currently accepted willingness to pay in the country (551,400,000 IR. Iran Rials (US$ 2,206) to 1,654,200,000 IR. Iran Rials (US$ 6,618)), the probability of TAVI cost-effectiveness against SAVR approximately lies within 0.31 to 0.83.

## Discussion

The cost difference between TAVI and SAVR is 368,180,101 I.R.I Rials (US$ 1,473), and the QALYs difference is 0.37. Also, the cost per QALYs is 995,081,354 I.R.I. Rials (US$ 3,980) for the base case analysis. The base case ICER is greater (~ 1.8 times the country’s GDP per capita as the considered WTP threshold in 2020). The probabilistic sensitivity analysis shows the mean ICER for the strategies is 981,765,302 I.R.I Rials (US$ 3,927) per QALY. In addition, the results of the one-way-sensitivity analysis demonstrate any change between 5 to 150% in stroke risk rate causes a range between 974,642,521 Iranian Rial (US$ 3,898) to 1,365,982,532 Iranian Rial (US$ 5,464) for the base case ICER. This amount of change for the permanent pacemaker implantation rate causes the base case ICER to vary between 973,762,098 Iranian Rial (US$ 3,895) to 1,320,762,751 Iranian Rial (US$ 5,283). This change in myocardial infarction rate leads the base case ICER to lie between 918,541,076 Iranian Rials (US$ 3,674) to 1,243,862,843 Iranian Rial (US$ 4,975). For atrial fibrillation, with the same range of rate change, the base case ICER will be between 975,832,032 Iranian Rial (US$ 3,903) to 1,216,768,043 Iranian Rial (US$ 4,867). Also, if this change considers in the rate of rehospitalization, the base case ICER will be between 942,870,541 Iranian Rial (US$ 3,771) to 1,175,435,431 Iranian Rial (US$ 4,702). The cost-effectiveness probability will be between 31 to 83% by varying the WTP thresholds.

The QALYs for TAVI are greater than SAVR. However, the major issue is related to the attributed costs to TAVI. TAVI is a more expensive procedure, the cost per patient for TAVI is 720,257,876 (US$ 2,881) and for SAVR is 352,077,775 (US$ 1,408), this means TAVI is two times costlier than SAVR. This cost difference can compensate for the greater QALYs advantage that is related to TAVI. In addition, it should be noticed that based on a meta-analysis the main advantage of TAVI in this regard is related to the first thirty days after the procedure, however, at one year follow up there is no difference in QALYs levels between the two procedures [29].

This is more important in a country such as Iran with economic and financial limitations over the past years. The fall of national exchange rates against other currencies (especially US$, and €) on one side has led to an excessive financial burden on the healthcare providers to use stents, mechanical or biological valves, and TAVI. On the other side, domestic economic challenges such as rising inflation rates have harmed the health system as well. These unprecedented impacts are more obvious in medicines and medical devices dependant procedures. Therefore, presumably, the TAVI procedure has had a hiking price as it includes technologies and devices (especially stents) that have inflated prices.

Fairbairn et al. in their economic evaluation in the UK have concluded that TVI is a dominant cost-effective procedure against SAVR even though its procedural costs make it costlier than SAVR. They also mentioned the post-hospitalization higher costs of SAVR in comparison with TAVI and subsequently the higher QALYs for TAVI vs SAVR [30]. This conclusion is not the same as what we have found for the Iranian elder population with a higher risk of AS. It seems their time horizon is ten years and by that time there are still alive patients (~ 15% of the cohort), but in our study, we have adopted a lifetime horizon and continue our calculations to the point that there is nobody left alive. In addition, a recent meta-analysis by Cao et al. has found that for patients at higher risk of surgery, the adverse outcomes including stroke, are not different between TAVI and SAVR. This is the same for myocardial infarction and acute renal failure. Generally, they have found that vascular complications are significantly higher in TAVI, but the bleeding is lower in TAVI-managed patients [31].

Another recently published Health Technology assessment (HAT) in Japan concluded that transfemoral TAVI (TF-TAVI) is a cost-effective procedure for managing the AS among high-risk patients that are not suitable candidates for SAVR [13].

There are other economic evaluations on using TAVI in managing AS patients, however, the comparators and population of interest are different from ours. In a Markov model economic evaluation by Goodall et al., the results present a 100% probability of cost-effectiveness in favour of TAVI against SAVR at €15,000 willingness to pay threshold. However, the results of this study come from AS patients at the intermediate risk in the PARTNER-II trial with a lower risk for participants [29]. Watt et al. estimated a likelihood of 100% being cost-effective for TAVI against medical management among AS patients who are not eligible for surgical intervention [32]. This study has used the PARTNER-I trial data, but for cohort B that means patients are not eligible for conventional surgery.

The important note in this regard is that these studies are from high-income countries that might not be faced with economic problems such as Iran’s specific context issues including paying a higher price for medical devices (here stents, heart valves, pacemakers, and angioplasty) due to sanctions and domestic mismanagement in allocating these resources for managing AS. This condition causes excessive costs that might be reflected in the cost-effectiveness analysis as well.

### Limitations

The main parameters for this model especially parameters about the survival rates have been extracted from the PARTNER trial (from the USA, Germany, and Canada), and other risks of post-surgery complications are from developed nations. It may be different from Iran. We tried to capture the impacts of these differences through a one-way-sensitivity analysis and changing the main drivers of base-case ICER, however, it may be still some uncertainties in this regard that only could be addressed through local studies.

Also, the country doesn’t have an explicit cost-effectiveness threshold (or WTP) and in the absence of a revealed WTP, we had to rely on the WHO’s recommendation by using GDP per capita as a basis for it, however, this approach may not demonstrate the actual opportunity cost in the country.

The study has been done in a situation where the country was exposed to substantial financial hardship, and the GDP per capita is one of the lowest over past years, it is quite probable that with changing the macroeconomic situation and subsequently changing in the GDP per capita, the results become different.

## Conclusion

TAVI doesn’t seem a cost-effective procedure for managing the high-risk AS among elders in Iran. This is so important when there is rigorous evidence supporting the cost-effectiveness of TAVI in that population for other countries. However, those countries are high-income countries with higher willingness to pay thresholds. The minimally invasive nature of TAVI and its impacts on the QoL of patients is still attractive enough to be considered for health insurers' attention. However, the Iranian policymakers need to consider the costlier aspect of the technology and the current economic situation of the country.

## Data Availability

All data and outputs are accessible through contacting the corresponding author.

## Abbreviations

AS: Aortic Stenosis
TAVI: Transcatheter Aortic Valve Implantation
SAVR: Surgical Aortic Valve Replacement
CVDs: Cardiovascular diseases
HVDs: Heart valvular disorders
QoL: Quality of Life
MIP: Minimally Invasive Procedure
ICER: Incremental Cost-Effectiveness Analysis
CEAC: Cost-Effectiveness Acceptable Curve
WTP: Willingness to Pay
NYHA: New York Heart Association
GDP: Gross Domestic Production
RCT: Randomised Controlled Trial
QALYs: Quality Adjusted Life Years

## References

[1] Organization WH. Cardiovascular diseases (CVDs) Geneva: World Health Organization; 2021 [Available from: https://www.who.int/news-room/fact-sheets/detail/cardiovascular-diseases-(cvds).

[2] Lopes MACQ, Nascimento BR, Oliveira GMM (2020). Treatment of aortic stenosis in elderly individuals in brazil: how long can we wait?. Arq Bras Cardiol.

[3] Ribeiro GS, Melo RD, Deresz LF, Dal Lago P, Pontes MR, Karsten M (2017). Cardiac rehabilitation programme after transcatheter aortic valve implantation versus surgical aortic valve replacement: systematic review and meta-analysis. Eur J Prev Cardiol.

[4] Lange R, Beckmann A, Neumann T, Krane M, Deutsch M-A, Landwehr S (2016). Quality of life after transcatheter aortic valve replacement: prospective data from GARY (German Aortic Valve Registry). JACC: Cardiovasc Interv.

[5] Bonow RO, Leon MB, Doshi D, Moat N (2016). Management strategies and future challenges for aortic valve disease. Lancet.

[6] Van Geldorp M, Heuvelman H, Kappetein AP, Busschbach J, Cohen DJ, Takkenberg J (2013). Quality of life among patients with severe aortic stenosis. Neth Hear J.

[7] Zelis JM, van't Veer M, Houterman S, Pijls NH, Tonino PA (2020). Survival and quality of life after transcatheter aortic valve implantation relative to the general population. IJC Heart Vasculature.

[8] Moore M, Chen J, Mallow PJ, Rizzo JA (2016). The direct health-care burden of valvular heart disease: evidence from US national survey data. ClinicoEcon Outcomes Res.

[9] Kodera S, Kiyosue A, Ando J, Komuro I (2018). Cost effectiveness of transcatheter aortic valve implantation in patients with aortic stenosis in Japan. J Cardiol.

[10] Shan L, Saxena A, McMahon R, Wilson A, Newcomb A (2013). A systematic review on the quality of life benefits after aortic valve replacement in the elderly. J Thorac Cardiovasc Surg.

[11] Shan L, Saxena A, Goh D, Robinson D (2019). A systematic review on the quality of life and functional status after abdominal aortic aneurysm repair in elderly patients with an average age older than 75 years. J Vasc Surg.

[12] Leon M, Smith C. The Partner Trial: placement of aortic transcatheter valve trial. USA: Clinical Trials Gov; 2010.

[13] Inoue S, Nakao K, Hanyu M, Hayashida K, Shibahara H, Kobayashi M (2020). Cost-effectiveness of transcatheter aortic valve implantation using a balloon-expandable valve in japan: experience from the Japanese pilot health technology assessment. Value in Health Regional Issues.

[14] Smith CR, Leon MB, Mack MJ, Miller DC, Moses JW, Svensson LG (2011). Transcatheter versus surgical aortic-valve replacement in high-risk patients. N Engl J Med.

[15] Leon MB, Smith CR, Mack MJ, Makkar RR, Svensson LG, Kodali SK (2016). Transcatheter or surgical aortic-valve replacement in intermediate-risk patients. N Engl J Med.

[16] Sarrafzadegan N, Mohammmadifard N (2019). Cardiovascular disease in Iran in the last 40 years: prevalence, mortality, morbidity, challenges and strategies for cardiovascular prevention. Arch Iran Med.

[17] Husereau D, Drummond M, Augustovski F, de Bekker-Grob E, Briggs AH, Carswell C (2022). Consolidated Health Economic Evaluation Reporting Standards 2022 (CHEERS 2022) statement: updated reporting guidance for health economic evaluations. Int J Technol Assess Health Care.

[18] Bank W. World Bank Country and Lending Groups Washington2022 [Available from: https://datahelpdesk.worldbank.org/knowledgebase/articles/906519-world-bank-country-and-lending-groups.

[19] Abdi Z, Hsu J, Ahmadnezhad E, Majdzadeh R, Harirchi I (2020). An analysis of financial protection before and after the Iranian health transformation plan. East Mediterr Health J.

[20] Makkar RR, Thourani VH, Mack MJ, Kodali SK, Kapadia S, Webb JG (2020). Five-year outcomes of transcatheter or surgical aortic-valve replacement. N Engl J Med.

[21] Daneshmand A, Jahangard E, Abdollah-Milani M (2018). A time preference measure of the social discount rate for Iran. Journal of Economic Structures.

[22] Swift SL, Puehler T, Misso K, Lang SH, Forbes C, Kleijnen J (2021). Transcatheter aortic valve implantation versus surgical aortic valve replacement in patients with severe aortic stenosis: a systematic review and meta-analysis. BMJ Open.

[23] Holland R, Rechel B, Stepien K, Harvey I, Brooksby I (2010). Patients' self-assessed functional status in heart failure by New York Heart Association class: a prognostic predictor of hospitalizations, quality of life and death. J Cardiac Fail.

[24] Fairbairn TA, Meads DM, Hulme C, Mather AN, Plein S, Blackman DJ (2013). The cost-effectiveness of transcatheter aortic valve implantation versus surgical aortic valve replacement in patients with severe aortic stenosis at high operative risk. Heart.

[25] Adam T. Making choices in health: WHO guide to cost-effectiveness analysis. Tehran: World Health Organization; 2003.

[26] National Accounts of Iran. Central Bank of Iran 2020. Available from: https://www.cbi.ir/page/2072.aspx.

[27] Iran Ministry of Health. Relative Value Unit for Medical and Health Services. Ministry of Health. Tehran: Mirmah Publishing Enterprise; 2017.

[28] Iran Ministry of Health. National tariffs for health and medical services. Tehran: Iran Ministry of Health; 2020.

[29] Goodall G, Lamotte M, Ramos M, Maunoury F, Pejchalova B, de Pouvourville G (2019). Cost-effectiveness analysis of the SAPIEN 3 TAVI valve compared with surgery in intermediate-risk patients. J Med Econ.

[30] Ando T, Takagi H, Briasoulis A, Grines CL, Afonso L (2019). Comparison of health related quality of life in transcatheter versus surgical aortic valve replacement: a meta-analysis. Heart Lung Circ.

[31] Cao C, Ang SC, Indraratna P, Manganas C, Bannon P, Black D (2013). Systematic review and meta-analysis of transcatheter aortic valve implantation versus surgical aortic valve replacement for severe aortic stenosis. Ann Cardiothoracic Surg.

[32] Watt M, Mealing S, Eaton J, Piazza N, Moat N, Brasseur P (2012). Cost-effectiveness of transcatheter aortic valve replacement in patients ineligible for conventional aortic valve replacement. Heart.

