# Correction: Transcatheter aortic valve implantation against conventional aortic valve replacement surgery in high-risk patients with aortic stenosis; a cost-effectiveness analysis

**DOI:** 10.1186/s13561-023-00420-3

**Published:** 2023-01-20

**Authors:** Hesam Ghiasvand, Shiva Khaleghparast, Naser Kachoueian, Kourosh Tirgarfakheri, Meysam Mortazian, Yaser Toloueitabar, Farhad Gorjipour, Seyran Naghdi

**Affiliations:** 1 Division of Health Sciences, Warwick Medical School, University of Warwick, Coventry, UK; 2 Cardiovascular Nursing Research Center, Rajaie Cardiovascular Medical and Research Center, Iran University of Medical Sciences, Tehran, Iran; 3 Department of Cardiac Surgery, Imam Hossein Educational Hospital, Shahid Beheshti University of Medical Sciences, Tehran, Iran; 4 Rajaie Cardiovascular Medical and Research Center, Iran University of Medical Sciences, Tehran, Iran; 5 AJA University of Medical Sciences Tehran Iran AJA University of Medical Sciences, Tehran, Iran; 6 Iranian Scientific Society of Extracorporeal Technology, Rajaie Cardiovascular Medical and Research Center, Iran University of Medical Sciences, Tehran, Iran; 7 National Center for Health Insurance Research, Tehran, Iran

**Correction: Health Econ Rev 13, 1 (2023)**

10.1186/s13561-022-00411-w

Following publication of the original article [1], the authors would like to correct the statement in Ethics approval and consent to participate section for the reason that the ethics code issuer is a Center that is affiliated with the Iran University of Medical Sciences, and not the University itself.

The statement currently reads:

This study has been approved by the Ethics Research Committee of Iran University of Medical Sciences.

The statement should read:

This study has been approved by the Ethics Committee of Rajaie Cardiovascular, Medical and Research Center.

The original article [1] has been corrected.
